# The Epileptic Colony at Chalcote

**Published:** 1901-07-27

**Authors:** 


### THE EPILEPTIC COLONY AT CHALCOTE.

ONLY a little time ago the fate of the epileptic was one of the saddest in the world. Men and women, perfectly sane, and, except at intervals, perfectly able to work, were outcasts of society, because at those intervals they were not only helpless but were objects of terror to those around them. The epileptic child cannot be received in an ordinary school, because of the effect which his occasional attacks may have on the sensitive nerves of his fellow-pupils. The epileptic man or woman cannot work regularly, and therefore often cannot obtain work at all. And yet work, above all healthy manual and if possible outdoor work, is the best possible occupation for epileptics. Fresh air and work which will lead to healthy physical fatigue, are most desirable, and these things are under ordinary conditions of life unattainable to all but a few. As the study of the disease showed what was the most hopeful and satisfactory system of treatment, those interested in epileptics began to plan how they could obtain for these sufferers the necessary conditions of amelioration and cure. This was the beginning of the epileptic colony. The largest of these is the German one at Bielefeld, and it is more or less on the lines of it that the English one at Chalcote St. Peter's is founded. It is maintained by the National Society for the Employment of the Epileptic, of which the Duke of Cornwall is President, and which has among its supporters many earnest friends of the afflicted—though, being as yet a comparatively new charity, it has plenty of room for more.

The colony was started in 1894, and in the first year of its existence gave a home and employment to twenty-four sufferers. Its members now number 136, but as it is computed that there are forty thousand epileptics in the United Kingdom, it will be seen that there is still plenty of work to do. The results already achieved are, however, very gratifying. The industries carried on are, first and chief, farming. The colony began with the possession of 135 acres of ground, but recently bought an adjoining farm, extending to 75 acres, part of which is pasture, but part a valuable cherry orchard. Work on the farm and in the garden gives employment to a considerable number of the colonists, and it is gratifying to record that the pecuniary results are satisfactory, the profit for the last year being £243. Building, carpentering, and other work of that kind is done for the colony by the inhabitants, and the profit from this department is £52 11s. 9d. During the winter months of last year, basket-making was introduced as a new industry, but on this a loss has to be recorded—a thing not much to be wondered at, as the workers were all inexperienced. The loss amounts to £4 12s. 5d. The women of the colony are employed in the laundry, in housework, or in needlework. It is to be hoped that the last is not the exclusive employment of any of the colonists, as we do not think that constant employment at such sedentary work can be good for epileptics.

From these figures, however, it will be seen that the colony is not one of those charities which one supports with a sigh, as simply tending to keep alive persons who are a burden to the world. In its five years of existence, it has shown that epileptics can be trained to be, at least to some extent, self-supporting. This is the more creditable since a large proportion of the colonists—35 out of 90 males, and 18 out of 44 females—followed no occupation before they came to the colony, and of the remainder few were engaged on work which they could follow there. They are beginners at whatever trade they now pursue, besides being hampered by grave bodily infirmity, accompanied in some cases by mental weakness. A certain amount of irregularity of employment must be expected from such workers, nor can any of them be accounted fit for the competition of the open labour market. In such an institution financial success is not the main object, nor should it be looked for, while at the same time it is gratifying to find that these afflicted ones can contribute in part to their own maintenance, while the work they do also forms a valuable part of their curative treatment. The report of the hon. medical staff states that "with the exception of a few individuals who would under any circumstances mentally deteriorate, as a natural consequence of their disease, the influence of the colony system as a remedial agent is beneficial. Its influence in this direction would appear to be due to the fact that it offers the means of employment to a class of the community who have difficulty in finding work on account of their malady; that it provides that kind of occupation which is most suitable for the nervous condition associated with this disease, and that, as the work usually performed is scheduled and under direct supervision, the least active and alert have a fair chance of success." The salutary influence of the life of the colony on the epileptic may be guessed from the fact that the "Dearmer" Home, which is intended for cases requiring isolation and special treatment has not required to be put into requisition.

It is to be regretted that the colony cannot extend its benefits to children, but the present regulations of the Act for the education of mentally defective and epileptic children render this impossible. The Act limits the number of patients at any home for epileptic children to fifteen, and forbids the aggregation of more than four such homes in any one place. The founders and supporters of the National Society for the Employment of Epileptics hold that the limitation of patients to fifteen in each home offers no particular advantage, and while protesting against any absolutely fixed standard of numbers, they think that about twenty-four inmates form the best establishment. It must be remembered that, proportionately, the expenses of administration and supervision decrease as the number of inmates rises. They reckon that this limitation of the number to fifteen would imply an increased cost of about £4 per child per annum. Moreover, the limitation of the total number of inmates to sixty is utterly opposed to the "colonial" idea, which aims at finding varied occupation for a large number of persons, of all ages, and reproducing, as far as may be among persons all suffering from a similar malady, the conditions of ordinary normal life. The intention of the limitation is obviously to prevent the disadvantages of making the colony of an institutional character; but it is contended, on the other side, that of the numerous epileptic colonies abroad, that at Bielefeld is the one which is the least institutional, and gives the fullest scope to individual character and development. Yet it is the largest, having a population of 1,500 epileptics of all ages. As an epileptic colony must be situated in the country, and cannot but be a place more or less apart socially from the rest of the world, the idea of those who favour the colonial idea is to make it in itself a little world, combining as great a variety of interests as possible.

